# Monitoring Chemical Processes Using Judicious Fusion of Multi-Rate Sensor Data

**DOI:** 10.3390/s19102240

**Published:** 2019-05-15

**Authors:** Zhenyu Wang, Leo Chiang

**Affiliations:** Continuous Improvement Center of Excellence, The Dow Chemical Company, Lake Jackson, TX 77566, USA; zwang12@dow.com

**Keywords:** process monitoring, sensor fusion, Bayesian approach, multi-rate measurements, spurious data, Industry 4.0

## Abstract

With the emergence of Industry 4.0, also known as the fourth industrial revolution, an increasing number of hardware and software sensors have been implemented in chemical production processes for monitoring key variables related to product quality and process safety. The accuracy of individual sensors can be easily impaired by a variety of factors. To improve process monitoring accuracy and reliability, a sensor fusion scheme based on Bayesian inference is proposed. The proposed method is capable of combining multi-rate sensor data and eliminating the spurious signals. The efficacy of the method has been verified using a process implemented at the Dow Chemical Company. The sensor fusion approach has improved the process monitoring reliability, quantified by the rates of correctly identified impurity alarms, as compared to the case of using an individual sensor.

## 1. Introduction

Initiated by the industrial internet of things (IIoT) concept, Industry 4.0 holds huge potential in improving operational effectiveness, developing new business models, and promoting sustainability [1]. With such a promising vista, Industry 4.0 has become a top priority for many companies, research centers and universities [2]. It in return facilitates the momentum of IIoT. As the number of instruments and sensors implemented in manufacturing processes increases, the amount of data collected also increases [3,4]. When a large amount of data, also known as big data, is available, it is non-trivial to apply relevant analytics methodologies to turn these data into useful and actionable information. 

In chemical processes, multiple sensors are employed to monitor key process variables, such as variables related to product quality and process safety. The sampling rates and the reliabilities of three typical sensors for monitoring chemical plants are given in Table 1. The Laboratory analyzer refers to instruments installed in a laboratory. Chemical samples are taken from the process stream (typically manually) and transferred to a lab for analysis. Because of the relatively clean environment, lab analyzers are considered to be the relatively reliable method for analyzing chemicals. However, its sampling frequencies are often too low for process monitoring and control purposes. The sample transfer process also introduces a time delay for the measurements, during which, the chemicals may degrade. Online analyzers are analytical instruments that are installed at the plant, not in a laboratory. The instrument is either in contact with the process or the sample is automatically delivered a short distance to the analyzer. They are also called pipe-centric analyzers, which clearly indicates the usual location of this type of analyzers. As the samples are taken in real-time from the process, the sampling frequency of online analyzer increases to once every several minutes. On the other hand, due to the limitation of the location, sometimes high temperature or high pressure, the online analyzer may not be the instrument with the highest accuracy and precision but be the one that can tolerate the tough environment. 

Software sensors are mathematical models that predict the infrequently measured product quality variables using frequently sampled inputs, such as temperature, pressure, and flow rate. This approach has been widely applied over past three decades [5], employing partial least squares (PLS) models [6,7], artificial neural networks [8,9,10], response surface models [11,12], and other data-driven and knowledge-driven mathematical models [13,14,15,16]. The sampling frequency is equivalent to the sampling frequency of the input data (temperature, pressure, etc). The reliability is high if the software sensor model is properly trained. However, the dynamics of chemical processes usually change over time, which is caused by factors like fouling, catalyst aging and operating condition changes. To account for changing process behaviors, soft sensors need to be updated periodically. Several online adaptive soft sensor algorithms have been proposed, including the Kalman filter-based method [17] and the means and variance update approach [18].

As given in Table 1, the accuracy of individual sensor can be impaired by a variety of factors, such as instrument malfunctioning, operator errors, and the inherent measurement errors. Reliability can be greatly improved using sensor fusion methodologies [19] using all the available data from different sensors. Applications of sensor fusion have been reported in a wide range of fields: Ernest and Banks explain how humans integrate vision and haptic information using a maximum-likelihood sensor fusion model [20]; Kim et al. predict vehicle location for safe lane changing by fusing radar and vision sensor data [21]; Li et al. combined visible and infrared images for remote sensing using robust principle component analysis and compressed sensing [22], Kim et al. present a multi-modal biometric recognition method based on convolutional neural network and score-level fusion [23]. A complete review of sensor fusion techniques, including classification, architecture and applications, was published by Castanedo [24].

For chemical processes, the application of sensor fusion is limited. Typically it is taken as a state estimation problem, in which the available measurements are used to correct the predictions by software sensors using a Kalman filter [25]. Based on the sequence of fusion and correction, the KF- based sensor fusion methodology can be classified into two classes: measurement fusion and track-to-track fusion (TTF) [26]. The former methodology fuses measurements from multiple hardware sensors first and utilize the fused value to correct the software sensor predictions [27]. This method has a lower error, but also a high computational cost. TTF utilizes multiple Kalman filters and multiple software sensors, one for each hardware sensor. It updates the individual sensor readings first and then fuses the updated values together. This results in sub-optimal state estimation but with reduced computing load. Harris and Gao [28] proposed an modified TTF method which performs better for dissimilar sensors system comparing to TTF. However like other abovementioned methods, modified TTF also assumes the measurements are sampled at the same rate and the all sensors are properly functioning. Fatehi and Huang [29] further improved the modified TTF to deal with multi-rate measurements with time delay, but accounting for differences in data quality or handling spurious data from different hardware instruments has not been considered. 

However, as discussed in Table 1, all sensors can make mistakes. To enhance the reliability of process monitoring, the sensor fusion methodology has to deal with spurious data. Presented in this paper is a multi-rate and judicious sensor fusion scheme based on Bayesian inference [30]. Comparing to our previous work [31], this new sensor fusion scheme is capable of dealing with multi-rate data. It can also identify and eliminate the spurious data from malfunctioning sensors. The proposed method has been examined in a chemical process utilized at Dow. The product impurity is inferred by fusing the measurements from a lab analyzer, an online analyzer and a PLS software sensor with different sampling rates. The software sensor was updated using mean and variance update algorithm to track the time-varying process dynamics. The online analyzer measurements were filtered to reduce their variability. These two sensor measurements were fused together with the lab measurements using maximize a posterior (MAP) approach [32]. It is shown that the sensor fusion approach improves the process monitoring reliability, quantified by the rates of correctly identified impurity alarm, comparing to the case of using an individual sensor.

The paper is organized as follows: the adaptive PLS soft sensor methodology based on mean and variance update is briefly introduced. Subsequently, the multi-rate judicious sensor fusion scheme derived via MAP is presented. The proposed approaches are then applied to a chemical process at Dow and its performances for monitoring the process quality variable are discussed. 

## 2. Adaptive PLS Soft Sensor

In this paper, PLS soft sensors are used to model the process quality variable using frequently measure process variables. The PLS soft sensor is given as follows:

(1) $$X=TP^{T}+EY=UQ^{T}+F$$

where $X\in R^{n\times m}$ and $Y\in R^{n\times p}$ are matrices of process and quality variables, respectively. Each element in the two matrices, $x_{ij}$ or $y_{ij}$, is mean-centered and scaled by standard deviation, i.e., $x_{ij}=(x_{0,ij}-\mu_{x_{0,j}}/\sigma_{x_{0,j}})$ and $y_{ij}=(y_{0,ij}-\mu_{y_{0,j}}/\sigma_{y_{0,j}})$, with $x_{0,ij}$ and $y_{0,ij}$ being the original process and quality values; $\mu_{x_{0,j}}$ and $\mu_{y_{0,j}}$ the means of the *j*-th process and quality variables; $\sigma_{x_{0,j}}$.and $\sigma_{y_{0,j}}$ the standard deviations of the corresponding variables. The T $\in R^{n\times m}$ and U $\in R^{n\times p}$ are the scores matrices for the X and Y matrices, while $P\in R^{m\times A}$ and $Q\in R^{p\times A}$ are the corresponding loadings matrices. By applying the PLS method, the process variables of m dimensions are projected to a reduced space of A (<m) dimensions, in which, the process variables are represented by the score vector, t. The number of principal components A is determined through cross-validation or information criterion [33,34]. The coefficients in the PLS soft sensor are estimated by:

(2) $$\beta_{PLS}=R\left(T^{T}Y\right)=RR^{T}XY$$

where R is the loading weight matrix following the notation in [35]. The prediction of the quality variables, $y\in R^{p}$, given the new set of scaled process variable $x_{s}\in R^{m}$ is given as:

(3) $$y=f\left(\beta_{PLS},\mu_{x},\sigma_{x},\;\mu_{y},\sigma_{y}\;\right)=\sigma_{y}\beta_{PLS}^{T}x_{s}+\mu_{y}$$

where the element in vector $x_{s}$ is again defined as $x_{j}=(x_{0,j}-\mu_{x_{0,j}}/\sigma_{x_{0,j}})$. The means and standard deviations, $\mu_{x},\sigma_{x},\;\mu_{y},\sigma_{y}$, are calculated using the training data.

To track the dynamics changes of chemical process, the PLS soft sensor here is updated periodically using the mean and variance update methodology [18]. When a new measurement is available, the coefficients, $\beta_{PLS}$ will remain the same, but the means and standard deviations, μ’s and σ’s given in Equation (3), of the process and quality variables will be updated. Then the new product quality variable is predicted by substituting the process variables and the updated means and standard deviations, $\mu_{x,new},\sigma_{x,new},\;\mu_{y,new},\sigma_{y,new}$, in Equation (3):

(4) $$y=f\left(\beta_{PLS},\mu_{x,new},\sigma_{x,new},\;\mu_{y,new},\sigma_{y,new}\;\right)$$

To minimize the computational burden, the soft sensor is updated only when significant process changes are detected. Two Key Performance Indicators (KPI’s), based on Hotelling’s $T^{2}$ and residuals, are used to quantify the changes. The scores, t, representing process variables in the reduced dimension space satisfies the Hotelling’s $T^{2}$ distribution, a generation of student t-distribution for multivariate analysis. Here we utilize the $T^{2}$ statistic to detect mean shifts of the process variables from PLS score vectors. The $T^{2}$ statistic is defined as:

(5) $$T^{2}=t_{0}^{T}\Lambda^{-1}t_{0}\sim \frac{A\left(n^{2}-1\right)}{n\left(n-A\right)}F_{A,n-A}$$

with $\Lambda =\frac{1}{n-1}T^{T}T$ defines the covariance of the scores t of the training dataset used to update the PLS sensor. The training dataset contains n samples. The $t_{0}$ is the score vector of the new process variable. The $F_{A,n-A}$ represents an *F*-distribution with A and n−A degree of freedom where A is the number of principal components. When the $T^{2}$ exceeds the upper limit, $T_{max}^{2}$, the means and standard deviations of the soft sensor model are updated. The $T_{max}^{2}$ is calculated using inverse Hotelling’s $T^{2}$ distribution with a pre-determined confidence level, 1−α:

(6) $$T_{max}^{2}=ginv\left(1-\alpha ,A,n-A\right)$$

In this paper, a 99.9% confidence level (α=0.001) is employed. The second KPI considered here is the absolute residual of the soft sensor prediction, e, which is determined as:

(7) $$e=\left|y-\hat{y}\right|$$

where y is the quality variable measured using lab instrument while $\hat{y}$ is the corresponding value predicted by the PLS soft sensor. The upper limit of the residual is calculated in a similar manner as given in Equation (6), but assuming a normal distribution of the residuals. As the raw values of two KPIs are easily affected by high frequency measurement noises, moving median filters are applied for improved reliability. Following the approach outlined in a previous publication [18], we use the filtering window size of 336 h. If the upper limit of the $T^{2}$-based KPI is exceeded, the update of means and standard deviations of the soft sensor will be processed, using the lab measurement (excluding the spurious ones) during the most recent *K* hours as y variable and corresponding process variables as x variable. Here we select K = 3000 which is the same value used in the previous paper [18]. In addition, one may consider rebuilding the soft sensor if the residual-based KPI exceeds the corresponding upper limit. This mean and variance update method works as effective as rebuilding PLS models at new operating conditions [18] when the subset of input variables affecting the output variables remains identical at the new operating conditions. 

## 3. Multi-Rate Judicious Data Fusion

In general, the fused measurement has smaller variance or higher precision as compared to individual sensor measurement. However, as discussed above, the individual sensor generates inaccurate measurements occasionally, called spurious data. When those values are fused together with other accurate individual measurements, the accuracy and precision of the fused measurement could be lower than the most accurate individual measurement. Therefore, the data fusion concept [30] must be judiciously applied to identify and eliminate the spurious data.

Assume that the individual sensor measurement is normally distributed around its true value, the probability of observing a senor measurement, ${\hat{y}}_{i,k}$, given the true value, $y_{k}$, is defined by:

(8) $$P({\hat{y}}_{i,k}|y_{k},\varphi_{i,k}=1)=\frac{1}{\sigma_{i,k}\sqrt{2\pi }}exp\left[-\frac{1}{2}{\left(\frac{{\hat{y}}_{i,k}-y_{k}}{\sigma_{i,k}}\right)}^{2}\right]$$

where the subscript i, k represents the *i*-th sensor’s measurement at time instant k. The $\varphi_{i,k}=1$ indicates that at the k**-th time instant, the corresponding sensor is working properly. The probability that a sensor is properly functioning defined in [30] is given by:

(9) $$\pi (\varphi_{k}=1|y_{k},{\hat{y}}_{i,k})=exp\left[-{\left(\frac{{\hat{y}}_{i,k}-y_{k}}{\alpha_{i,k}}\right)}^{2}\right]$$

with the value parameter $\alpha_{i,k}$ assumed as:

(10) $$\alpha_{i,k}^{2}=2\sigma_{i,k}^{2}\prod_{j\neq i}^{S}m^{2}/\prod_{j\neq i}^{S}{\left({\hat{y}}_{j,k}-{\hat{y}}_{i,k}\right)}^{2}$$

where *m* is selected as the largest divergence allowed between the sensor measurements. When the *i*-th sensor reading is close to other sensors readings, the value of $\alpha_{i,k}^{2}$ will be larger. Therefore, the probability that this sensor is properly functioning, as given in Equation (9), will be larger. It can be also seen later in this section in Equation (16), with a larger $\alpha_{i,k}^{2}$ value, the corresponding sensor data will have smaller weight in the fused value comparing to the data from other more reliable sensors. In this paper, *m* is selected as 50% of the largest measurement at time instant *k*. This value is selected based on the historical data of the sensors. If the data from different sensors are close to each other, the value of m can be smaller. If the sensors readings usually have a larger deviation, then a larger m should be selected. The variance for each sensor, ${\hat{\sigma }}_{i,k}^{2}$, is estimated as:

(11) $${\hat{\sigma }}_{i,k}^{2}=\frac{1}{L}\sum_{j=k-L+1}^{k}{\left({\hat{y}}_{i,j}-{\bar{y}}_{i,k}\right)}^{2}$$

The average value for the *i*-th sensor measurement, ${\bar{y}}_{i,k},$ is calculated using L most recent measurements, i.e., ${\hat{y}}_{i,k-L+1}$, ${\hat{y}}_{i,k-L+2}$… ${\hat{y}}_{i,k}$. In this paper, we select a relative short 48-hour time period. That means for lab measurement, L=4 while for online analyzer and software sensor, L=48. The smaller window size leads to higher variance when a sensor reports an alarm. Because the proposed sensor fusion method assigns more weight on the sensor readings with smaller variance, the smaller window size will make the identification of false alarm easier, especially when there are only two sensors available. 

For a properly functioning sensor, the deviations of its reading from other sensors readings should be always smaller than the allowed deviation, *m*. By Equation (10), the following constraint for a properly functioning sensor should be satisfied:

(12) $$\alpha_{i,k}^{2}\geq 2{\hat{\sigma }}_{i,k}^{2}$$

However, when a sensor is malfunctioning, its reading will deviate significantly from other sensor readings. This will lead to a smaller $\alpha_{i,k}^{2}$ value and violate the above constraint. The fused value will only take the reliable measurements satisfying inequality (12) into account.

Given the measurements from the three independent sensors mentioned previously, the posterior probability of the true product quality variable is given by:

(13) $$P\left(y_{k}|{\hat{y}}_{i,k},\varphi_{i,k}=1\right)=\frac{P\left(y_{k}\right)\prod P({\hat{y}}_{i,k}|y_{k},\varphi_{i,k}=1)\pi \left(\varphi_{i,k}=1\right)}{\prod P\left({\hat{y}}_{i,k}\right)\pi (\varphi_{i,k}=1|y_{k},{\hat{y}}_{i,k})}$$

for i=1, 2, 3. The optimal estimation of the actual product quality variable value, $y_{k}$, via maximize a posterior (MAP) approach [32] is given by:

(14) $$y_{k}=\sum_{i=1}^{S}\frac{\frac{1}{2\sigma_{i,k}^{2}}-\frac{1}{\alpha_{i,k}^{2}}}{\sum_{j=1}^{S}\left(\frac{1}{2\sigma_{i,k}^{2}}-\frac{1}{\alpha_{i,k}^{2}}\right)}{\hat{y}}_{i,k}$$

The estimation of the product quality variable given in Equation (14) can be interpreted as a weighted average of predictions by three sensors and is given by:

(15) $${\hat{y}}_{k}=w_{1,k}{\hat{y}}_{1,k}+w_{2,k}{\hat{y}}_{2,k}+w_{3,k}{\hat{y}}_{3,k}$$

The weight coefficients of the *i-th* sensor reading at time instant *k* is then calculated as:

(16) $$w_{i,k}=\left(\frac{1}{2\sigma_{i,k}^{2}}-\frac{1}{\alpha_{i,k}^{2}}\right)/\sum_{j=1}^{3}\left(\frac{1}{2\sigma_{i,k}^{2}}-\frac{1}{\alpha_{i,k}^{2}}\right)$$

for i=1, 2, 3. As the variance of the sensor measurement and the deviation of a sensor from other sensors data decreases, the weight coefficients increase and the corresponding sensor measurements have more contribution to the fused estimate ${\hat{y}}_{k}$. 

The above results are based on the case that all three sensors are available. As the lab analyzer has a lower sampling frequency compared to the online analyzer and the soft sensor, in most of cases, there are only two sensors data available. In such a case, the malfunctioned sensor is not that easy to identify. Fusing a spurious sensor data to the other accurate sensor data may corrupt the accurate one. The variance of the posterior distribution for the fused value of two sensor readings can be derived from Equation (13) and given by:

(17) $$\sigma_{f}^{2}=\frac{\sigma_{i,k}^{2}\times \sigma_{j,k}^{2}}{\sigma_{i,k}^{2}+\sigma_{j,k}^{2}}\times \frac{m^{2}}{m^{2}-{\left({\hat{y}}_{j,k}-{\hat{y}}_{i,k}\right)}^{2}}$$

To ensure an improved precision or decreased variance, the posterior variance $\sigma_{f}^{2}$ must be smaller than the prior variance of the two individual sensors, i.e.,$\;\sigma_{f}^{2}\leq min\left(\sigma_{i,k}^{2},\;\sigma_{j,k}^{2}\right)$. Otherwise, the two sensors value will not be fused and the sensor measurement with smaller variance will be used. 

## 4. Proposed Sensor Fusion Scheme

**Figure 1:**
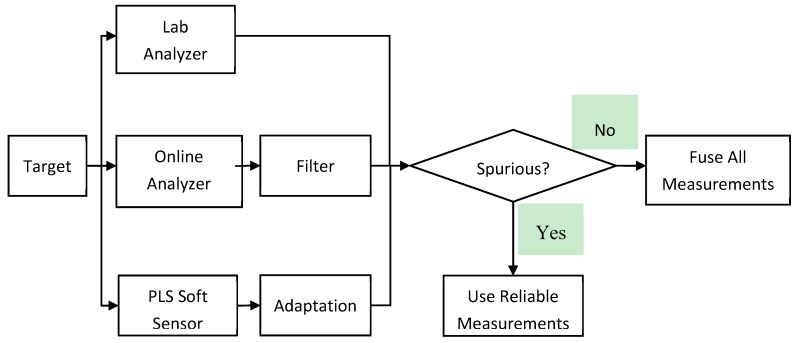
Proposed multi-rate and judicious sensor fusion scheme.

The proposed multi-rate and judicious sensor fusion scheme is shown in Figure 1, while the steps for executing the proposed sensor fusion method are summarized in Table 2. Three sensors, Lab Analyzer, Online Analyzer and Software sensor model are monitoring the sample variable. The online analyzer is filtered to reduce the measurement variance. The software sensor model is updated periodically to track the changes in process behaviors. When the lab measurement is available, the data from all three sensors are compared to check if any of them is spurious. The spurious data will be eliminated and the fused value is calculated using the reliable data and the above equations. When the lab measurement is not available, the spurious data is identified by comparing the data from the online analyzer and the software sensor. If either of them is spurious, the reliable data will be used as the fused value. Otherwise, the fused value is determined based on data from both sensors. Using the proposed methodology, multi-rate sensor data can be used to improve the process monitoring accuracy and reliability. The malfunctioning sensor can be identified and eliminated from the fusion process.

## 5. Results and Discussion

**Figure 2:**
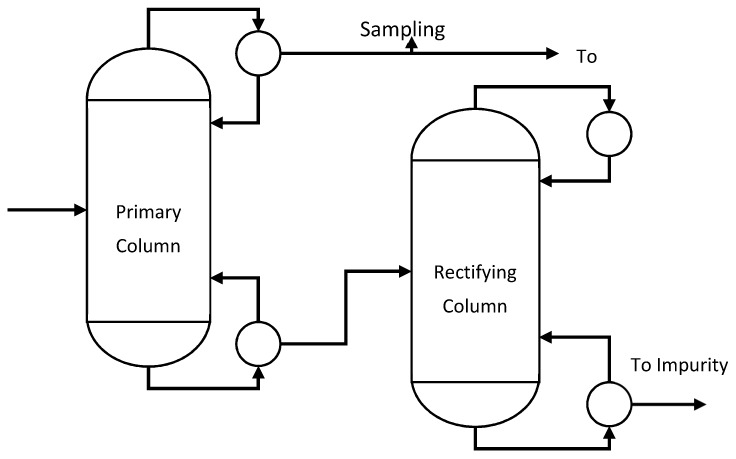
Process flow diagram of the refining section of the production process.

The proposed sensor fusion scheme is applied to a chemical process consisting of two distillation columns at Dow. The flow diagram of the process is shown in Figure 2. The product quality variable, namely the product impurity, is measured by both online and lab analyzers at the outlet of the primary column, marked as Sampling Point. Besides the impurity, input variables such as temperature, pressure, reflux ratio, etc., are measured and interpolated hourly to develop the soft sensor model. The lab analyzer sampling the impurity in every 12 h, while the online analyzer and soft sensor data are available in every 1 h. 

The product impurity is a key quality variable that must be accurately monitored. Misidentification of off-spec products will lead to significant economic loss to both the company and the consumer. On the other hand, over-reaction to false alarms, i.e., mistaken eligible product to off-spec ones, will cause unnecessary operational costs. Therefore, using sensor fusion methodology to improve the monitoring precision and reliability is essential for improving the efficiency and sustainability of manufacturing processes.

In the following sub-sections, we first discuss about the preprocessing for the data from three sensors considered in the proposed scheme: the software sensor is developed using the lab data as the output variable and is updated when the $T^{2}$ based KPI exceeds its upper limit; the online analyzer data is filtered by a moving median filter with a properly tuned window size. Then we fuse all the multi-rate data to infer the value of product impurities. Examples of handling spurious data from individual sensor, such as false impurity alarm, are discussed as well.

## 6. Adaptive PLS Soft Sensor

**Figure 3:**
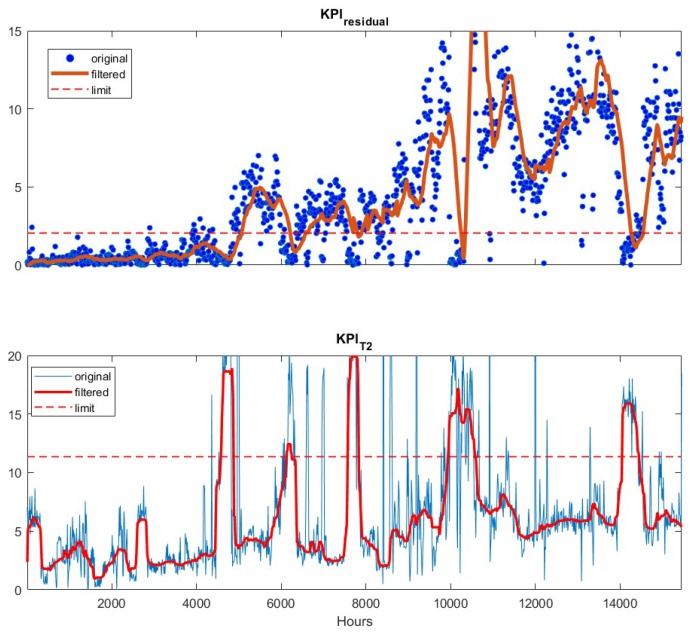
Residual-based KPI (upper) and $T^{2}$-based KPI (lower) for static software sensor without adaptation, applied to the testing data. The raw KPI values (●/─), the filtered values (─) and the upper limits (--) are depicted as well.

We first examine the mean and variance update algorithm [18] for developing an adaptive soft sensor. The original PLS soft sensor including nine process variables was developed using data from four years ago. The data and model development are not discussed here. Rather, we use the recent two years data as testing data to examine how accurately the original model without adaptation predicts the product impurity. The resulting KPIs are plotted in Figure 3. The residuals between the soft sensor predictions and the lab measurements are shown in blue dots in the upper figure. As the lab measurement is in every 12 h, the residuals are sparse comparing to the $T^{2}$ plot given in the lower sub-figure. The calculation of $T^{2}$ is based on the frequently measured input variables, pressures, temperatures and flow rates, so it is available every hour and is plotted in continuous line in Figure 3. As is seen in the figure, both the residual (upper figure) and $T^{2}$ (lower figure) based KPIs exceed the corresponding upper limit (dashed lines) after 4000 h. This indicates that the soft sensor needs to be updated to ensure accurate predictions.

**Figure 4:**
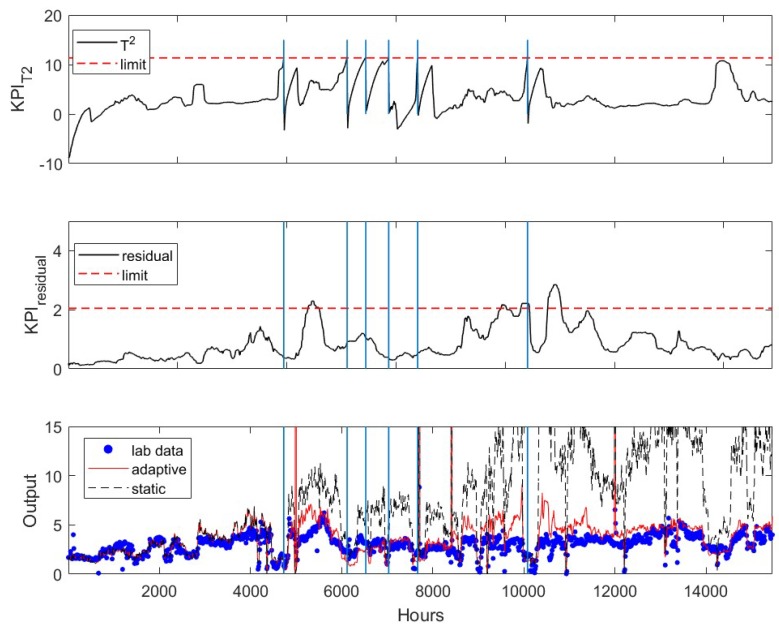
Evolution of residual-based (top) and $T^{2}$-based (middle) KPI’s of adaptive software sensor applied to the testing data set. Comparison of static (--) and adaptive (─) software sensors predictions with lab (●) measurements (bottom). Vertical lines indicate when the model was updated.

After the application of the mean and variance update algorithm, the $T^{2}$-based KPI (top sub-figure in Figure 4) first exceeds its upper limit at *k* = 4733, while the residual-based KPI (middle figure) is still under its upper limit. The bottom plot in Figure 4 shows the prediction of the updated soft sensor (red line) after updating the soft sensor, which tracks the lab measurements (blue dots) much closer than the original soft sensor (black line). The accuracy of the original soft sensor decreases significantly over time. In total, the soft sensor updates based on the criteria outlined in the earlier section are activated six times during the period examined here. The time instances when the software sensor are updated are marked by the vertical lines. It has shown that the adaptive model accurately track the product impurity. The residual-based KPI slightly exceeds the upper limit twice. This may indicate that the accuracy of the prediction can be further improved with a rebuilt soft sensor, which is not discussed in this paper. 

## 7. Tuning the Moving Median Filter for the Online Analyzer

**Figure 5:**
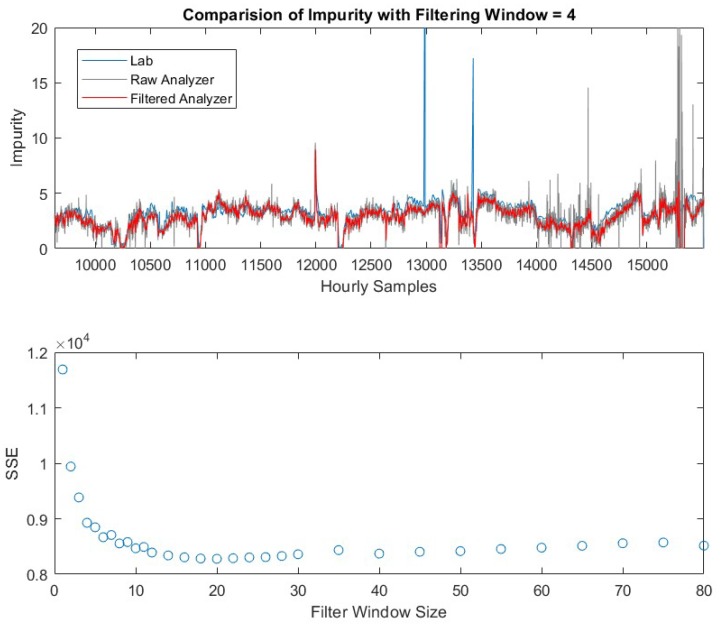
Comparison of hard sensor measurements (upper). Sum of Squared Error vs. different filtering window sizes (lower).

The online analyzer provides frequent measurements for the variable of interest, but with a relatively large variance. In addition to that, the online analyzer also provides spurious values during *k* = 15170 to 15250 due to sensor malfunctioning as shown in Figure 5. Therefore, a moving window median filter is applied to reduce the variability of the measurements. The filtered output is the median value of the past N measurements. The window size N should be appropriately selected to reduce the variability and preserve the sensitivity to detect the abnormal events. Here we use the lab measurements as the reference and select the optimal value of *N* to minimize the sum of squared error (SSE) between the lab measurements and the filtered online analyzer measurements. The filter size considered here is in the range of 1 to 80. As shown in the lower figure of Figure 5, the SSE decreases as the window size increases from 1 to 14. The SSE obtained at *N* = 1 is the exact SSE of the raw online analyzer measurements. It can be seen as the window size of the filter increases, SSE has been reduced, with an optimum window size of approximately 18 for this data set. 

On the other hand, the filtered measurements should capture the events when the product impurity exceeds its upper limit at 7.5. As shown in upper figure of Figure 5, such an event (impurity alarm when the concentration is greater than 7.5) occurs at *k* = 11,996 to 12,001. As the filter size increases, the filtered values at these time instants decrease (not shown in this paper). Therefore, the optimal size *N* is determined by the trade-off between minimizing SSE and maintaining the detection of impurity alarm. Here we select a filter window size of *N* = 4. The resulting filtered online analyzer measurements (red) are compared with the raw measurements (grey) in the upper graph of Figure 5. It has seen that the variability of the filtered values has been significantly reduced and the impurity alarm has been detected as well.

## 8. Fusion of Three Sensors 

Due to the availability of historical data from the online analyzer, we utilize the data of all three sensors during the time period of *k* = 9700 to 15,420. Each sensor reports multiple impurity alarms during this time period and the corresponding time instances are listed in column 1 of Table 3. However, only one impurity alarm at *k* = 11,996–12,001 is verified as a true one by the plant engineers, while others are confirmed as false alarms. The true alarm is denoted with “T” in column 2 of Table 3 while the false ones are marked with “F”. The robustness of the sensor measurements is quantified by the rate of correctly identified alarms, including the miss of detection for true alarms and false alarm rate, defined as number of true alarms missed (false alarm reported) per year. These two indicators are given in the last two rows of Table 3. The efficacy of the sensor fusion approach will be verified if the two indicators are improved compared to individual sensors.

**Figure 6:**
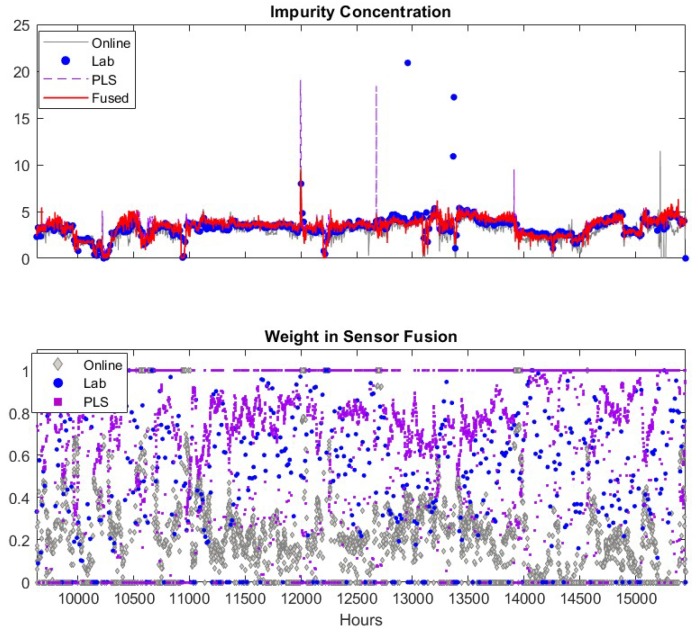
Fusion of filtered online analyzer data with other sensors. Upper figure: lab data (●), filtered online analyzer data (─), Adaptive PLS software sensor data updated as shown in Figure 4 (--) and fused value (─). Lower figure: Weights of lab data (●), online analyzer data (♦), PLS software sensor data (■).

As discussed previously, we select a window size of 48 h for estimating the measurement variance in Equation (14) and the maximal allowed deviation between sensors as 50% of the highest sensor reading. One may select other values based on the specific application. Results from fusing the adaptive soft sensor predictions, the lab analyzer measurements and the filtered online analyzer measurements are shown in the upper plot of Figure 6. 

The measurements of the online analyzer, the lab analyzer and the soft sensor are in general close to each other. However, each of these sensors has false alarms (product impurity > 7.5). The lab measurements have two false alarms at about k = 12,957 and 13,365. These false alarms may be caused by human error when recording the data into database, but it is a good test for the sensor fusion scheme as the lab measurement is usually assigned highest weight in the fusion. If the fusion scheme can detect and eliminate the spurious lab data, the efficacy and robustness of the propose scheme is confirmed. The prediction of the adaptive soft sensor has two false alarms at k = 12,676 and 13,910. The filtered online analyzer data contains just one false alarm at *k* = 15,190. The fused measurements captures the true impurity alarm at *k* = 11,995, and generates no false impurity alarms. In addition, the fused values in general follow the trend of the three sensors. Therefore, the fused measurements are superior to the measurements of the individual sensors. In the lower panel of Figure 6, we depicted the weight coefficients of each data source. According to the weight coefficients, the lab measurements and the PLS predictions usually have large contributions (larger weight values shown in the figure) to the fused values while the influence of the online analyzer is usually small. This is because the online analyzer measurement usually has more frequent variations compared to other sensors which results in a larger variance for the measurement. In addition, when the soft sensor becomes less accurate, the corresponding weights decrease significantly and the fused values are mainly determined by lab measurements. Similarly, when the lab analyzer offers false alarm at *k* = 12,957 and 13,365, the corresponding weights drop to relative low values. These observations verify the efficacy of the proposed sensor fusion methodology presented in this paper. 

We summarize the capability of the individual and fused sensors for detecting impurity alarms in column 3–7 of Table 3. The symbol “✔” indicates the corresponding sensor reports an alarm. For example, the true alarm at *k* = 11,996–12,001 is detected by all sensors. In this table, we can tell that the lab analyzer, usually being considered as the most reliable process monitoring method, gives two false alarms. The adaptive soft sensor also gives two false alarms. The online analyzer reports seven false alarms in the period of k = 15,161–15,222 caused by sensor malfunction. The filtered online analyzer only provides one false alarm. The fused sensor detect the true alarm without giving any false ones. Therefore, the fused sensor has the smallest false alarm rate compared to other sensors.

**Figure 7:**
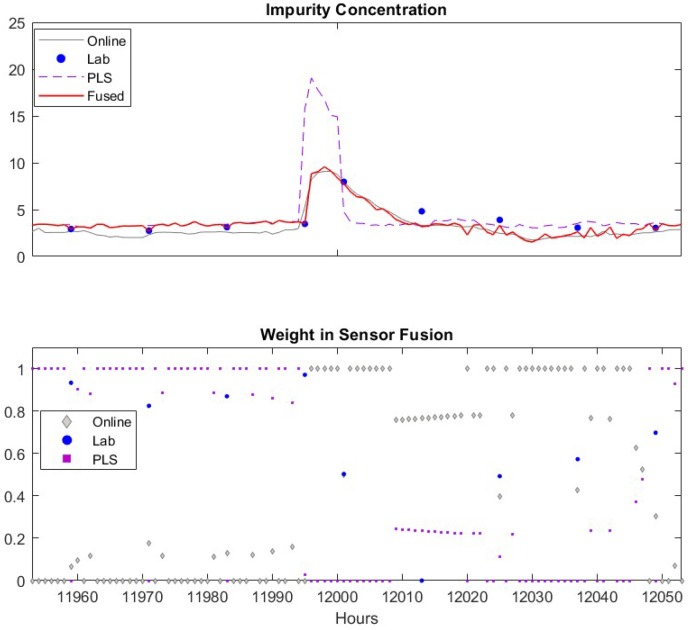
Sensor readings of quality variable around when the true alarm occurs. Upper figure: Comparisons of lab data (●), online analyzer data (─), PLS software sensor data (--) and fused value (─). Lower figure: Weights of lab data (●), online analyzer data (♦), PLS software sensor data (■).

**Figure 8:**
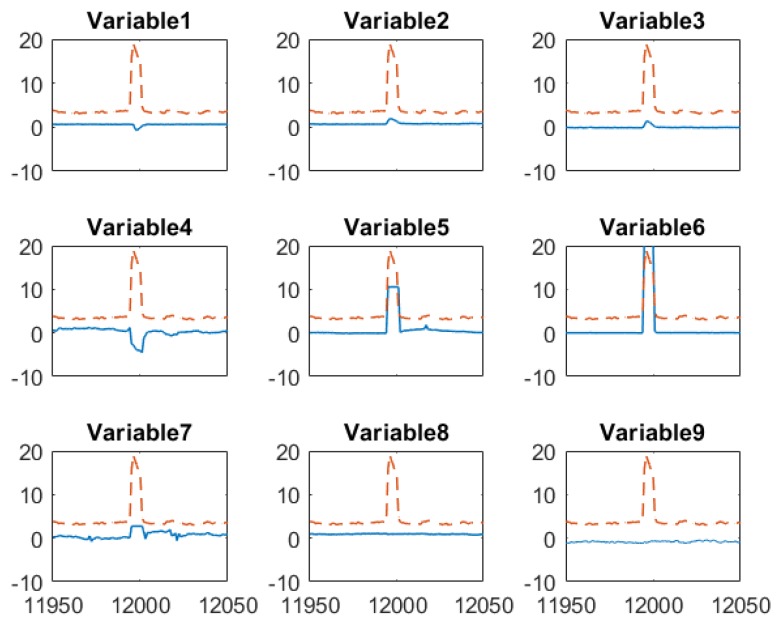
Mean-centered and scaled input variables (--) of the software sensor and soft sensor predicted impurity (─) around when the true alarm occurs.

To gain more insights about how the proposed sensor fusion scheme handles spurious data, we zoomed in three representative time periods, k = 11,950–12,050, k = 12,630–12,730 and *k* = 13,330–13,430. In the first period, all sensors detect the true alarm while in the rest two periods, software sensor and lab measurement provide false alarms, respectively. The individual sensor readings of the quality variable as well as their corresponding weights are plotted in Figure 7. The PLS sensor first provide an alarm at *k* = 11,995, one hour ahead of the online analyzer. In contrast, the analyzer and lab measurement obtained at *k* = 11,995 show only slightly increase in impurity level. As both online analyzer and soft sensor provide alarm after *k* = 11,996, an additional lab measurement was taken at *k* = 12,001, which confirms the impurity level does increase exceed the specification limit. Because the soft sensor shows much higher impurity value during the true alarm compared to other sensors, its weight in the fused value is zero. The fused value depends on online analyzer and lab measurements. The nine input variable values of soft sensor during this period are plotted in Figure 8. It has shown that the alarm by the soft sensor is caused by the increased value in Variable 1–7 during *k* = 11,996–12,001.

**Figure 9:**
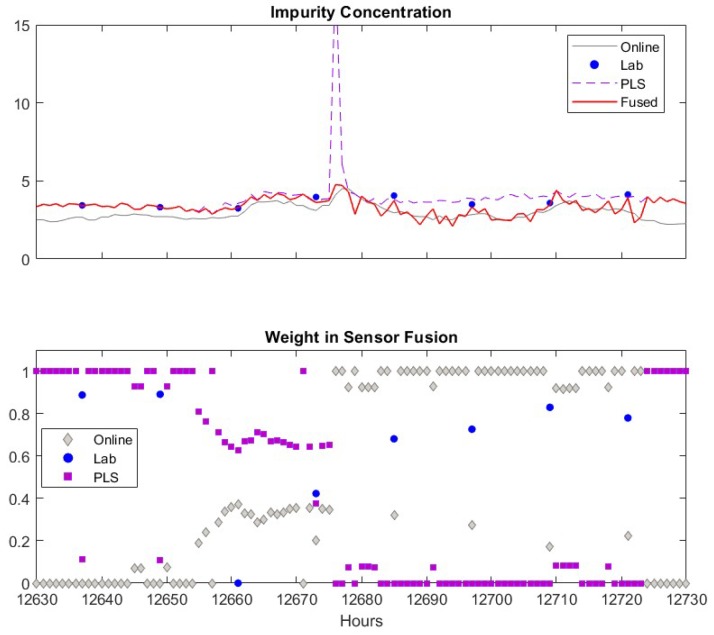
An example when PLS software sensor provides spurious data. Upper figure: Comparisons of lab data (●), online analyzer data (─), PLS software sensor data (--) and fused value (─). Lower figure: Weights of lab data (●), online analyzer data (♦), PLS software sensor data (■).

**Figure 10:**
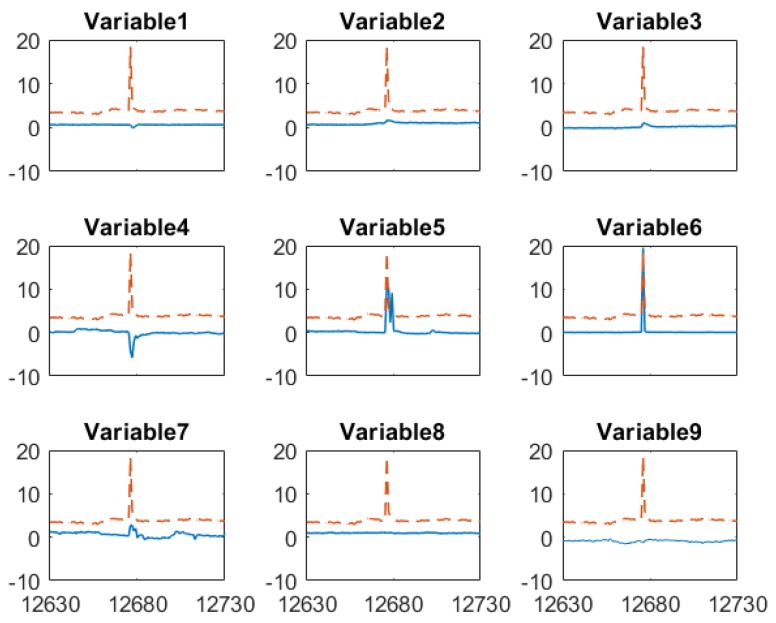
Mean-centered and scaled input variables (--) of the software sensor and soft sensor predicted impurity (─) around when the false alarm by PLS soft sensor occurs.

An example how the fusion scheme handling false alarm from the PLS software sensor is given in Figure 9. The false alarm by the software sensor shown in Figure 9 results from the sensor measurements of several input variables in the PLS model as shown in Figure 10. Similar to the case of true alarms, process Variable 1–7 increases when the false alarm reported by the software sensor. However, most of these high values only last for two hours. This indicates the abnormal conditions in the process didn’t last long enough to generate sufficient impurities to increase the impurity concentration beyond the specification limit. In addition, the online analyzer reading did increase from 3.9 to 4.6, which confirms there was increase in impurity level. By comparing the soft sensor readings in this false alarm and in the above true alarm, we can tell that the PLS sensor provide alarms when operating conditions change, but such changes may not last long enough to generate off-spec impurity concentrations. In such a case, the PLS soft sensor will report false alarms, but the proposed sensor fusion method can recognize the inaccurate prediction from the PLS soft sensor. 

We now focus on discussing how the proposed method detects spurious sensor readings or false alarms. When the software sensor shows a false alarm (the peak in the upper sub-figure), the fused value weighs the online analyzer heavily and assigns almost zero weight to the soft sensor. This is because that the variance of the software sensor significantly increases due to the rapid change of its measurement. Meanwhile, the variance of the online analyzer remains at a similar low value. As the moving window size for calculating the variance is 48 h, the spurious data will cause a high variance of the software sensor data for the next 48 h. As a result, the weight of software sensor remains low until 48 h later, when the previous spurious data point is “forgotten” by the algorithm. During the period of k = 12,630–12,650, the software sensor predictions are very close to the lab measurement, as given in the upper sub-figure, and, as shown in the lower sub-figure, the weights of software sensor data are almost 1 when there is no available lab measurement, which means the fused inference solely depends on the software sensor data. When the lab measurement is available, it can be seen that the fused value relies on 90% of the lab and 10% of the software sensor data. As the online analyzer data deviates from two other measurements, the weight of the online analyzer is almost zero during this period. As the deviation of online analyzer from two other sensors decreases, the weights of the online analyzer increase. 

**Figure 11:**
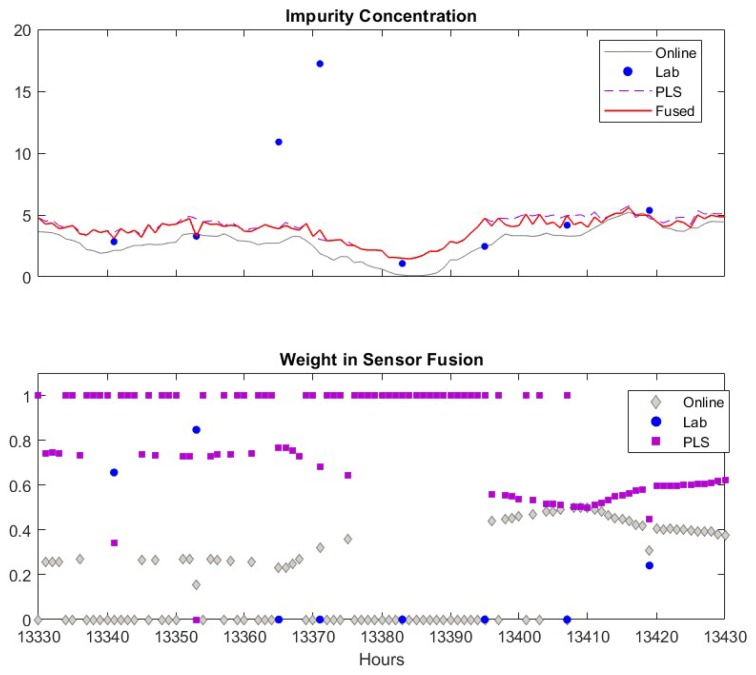
An example when lab data are spurious. Upper figure: Comparisons of lab data (●), online analyzer data (─), PLS software sensor data (--) and fused value (─). Lower figure: Weights of lab data (●), online analyzer data (♦), PLS software sensor data (■).

A similar case that lab analyzer gives spurious data is shown in Figure 11. Again, the software sensor in general has higher weight compared to the online analyzer. When the lab data are available, the fuse value largely depend on the lab data. However, the weight of lab data drops zero when false alarms given by the lab measurements. It has shown that during *k* = 13,375–13,395, the fused value solely depends on the PLS software sensor values, despite the readings from the soft sensor and the online analyzer are close to each other. This is because that the variance of the fused value calculated using Equation (17) would be larger than the variance of the value from the soft sensor. In such a case, the values from the online analyzer and the software sensor are not fused together. As the soft sensor and online analyzer measurements become closer afterwards, the fused values rely on both of them.

Using the above examples, we have explained that how the proposed sensor fusion scheme identifies and eliminates the spurious data or false alarms. The proposed method may be challenged when two of the three sensors provide spurious data at the same time. However, it is not likely to happen, because the factors affecting the sensor accuracies are different. In addition, even if such case did happen, the sensor readings are expected to be obviously different. And, extra lab sample will help to confirm true product impurity value. 

## 9. Conclusions

In this paper, we have proposed a sensor fusion scheme to combine multi-rate measurements from both hardware and software sensors. It is capable of identifying and eliminating the spurious data from individual sensors to produce a highly reliable soft sensor. As a result, the reliability and precision of monitored variables related to product quality and process safety will be improved. 

The proposed scheme utilizes three typical sensors for monitoring a chemical process: software sensor, online analyzer, and lab analyzer. Due to the time-varying dynamics of the chemical processes, a mean and variance update based adaptation algorithm is applied to keep the soft sensor tracking the process changes. The measurements of the online analyzer are filtered using a moving median filter to reduce its variability. Then the measurements from the adaptive soft sensor, the online analyzer and the lab analyzer are then fused using Bayesian inference.

We have examined the sensor fusion scheme reported in this paper using a chemical process implemented at Dow for monitoring the product impurity. It has demonstrated that the fused estimation of the product impurity is more reliable and accurate than the measurement of any individual sensor. Specifically, the fused estimation follows the trends of the measurements by the individual sensors, while only reporting the true impurity alarm without any false alarms in contrast to the individual sensors.

## Figures and Tables

**Table 1:** Typical hard and soft sensors applied to monitoring chemical processes.

Sensor Type	Laboratory Analyzer	Online Analyzer	Software Sensor
**Sampling Frequency**	Hours	Mins/Seconds	Mins/Seconds
**Reliability**	Highest	High	High
**Factors Affecting Accuracy**	Instrument error Time delay Operator error	Instrument error Detection limit Location	Plant-model mismatch Plant behavior change Input measurement error

**Table 2:** Steps of the Proposed Sensor Fusion Method.

Step	
1	At time instant *k*, check if software sensor needs an update by calculating $T^{2}$ using Equation (5). If $T^{2}>T_{max}^{2}$, go to Step 2, otherwise, go to Step 3.
2	Update the mean and standard deviation using past *K* lab measurement (excluding the spurious lab measurements) as *y* variable and corresponding process variables as *x* variable.
3	For available sensor readings, calculate $\alpha_{i,k}^{2}$ and $\sigma_{i,k}^{2}$ using Equation (10) and Equation (11), respectively. Select the sensor readings that satisfy constraint (12)
4	Fused the selected sensor readings using Equation (14).
5	If only two sensors data available, calculate $\sigma_{f}^{2}$ using Equation (17). When $\sigma_{f}^{2}\leq min\left(\sigma_{i,k}^{2},\;\sigma_{j,k}^{2}\;\right)$ fuse the two sensor readings. Otherwise, take the reading with smaller $\sigma^{2}$ as the final reading.
6	Repeat step 1–5 for time instant *k* + 1

**Table 3:** Comparison of Sensors’ Detections for Impurity Alarms.

Index, *k*	T/F	Lab Analyzer	Online Analyzer (Raw)	Online Analyzer (Filtered)	Adaptive PLS	Fused Sensor
11,996–12,001	*T*	✔	✔	✔	✔	✔
12,676	F				✔	
12,957	F	✔				
13,365	F	✔				
13,910	F				✔	
14,376	F		✔			
14,381	F		✔			
15,161–15,222	F		7x✔	✔		
15,320	F		✔			
Missed True Alarm		0	0	0	0	**0**
False Alarm Rate		1.1/year	5.6/year	0.6/year	1.1/year	**0**
